# Hospital Meetings

**Published:** 1894-02-24

**Authors:** 


### Hospital Meetings.

### CHARING CROSS HOSPITAL.

The annual general court of Governors was held on February 21st at Charing Cross Hospital, with Mr. J. B. Martin, the Treasurer of the hospital, in the chair, among those present being Dr. Watt Black, Rev. W. McCarthy, and Messrs. Barrett, H. S. Samuel, Park, and Nightingale. In the report presented to the Governors, especial stress was laid upon the unsatisfactory financial condition of the institution, the ordinary income of the year having slightly decreased, while the number of patients was, on the contrary, larger—the out-patients numbering 23,001, and the in-patients 2,503. The Chairman, in his introductory statement, spoke of the grave loss it would be to the district were the hospital to be wholly or even partially closed, as well as the blow such a closing would be to the voluntary hospitals in general, affording, as it would, a handle to the enemies of the present system. Nevertheless, deeply as he regretted the necessity, the hospital was already so much in debt that unless their exertions to procure the necessary funds were successful, he saw no other alternative than to reduce the number of beds at present available. The Chairman further stated that after considerable trouble a site for a Convalescent Home had at length been secured near Limpsfield Common, thanks to the generosity of Mr. Passmore Edwards, and that among the improvements necessitated during the past year was an increase in the accommodation for the nursing staff. With a hearty vote of thanks to the Chairman the proceedings then closed.

### METROPOLITAN CONVALESCENT INSTITUTION.

The annual general meeting of this institution was held at 32, Sackville Street, on February 20th, with Mr. J. R. Wade in the chair. The report for 1893 stated that altogether 5,276 patients had been treated at Walton-on-Thames, Bex hill-on-Sea, and the Children's Home at Herne Bay, in spite of the fact that on account of scarlet fever no patients were for some time admitted into the latter. The subscriptions and donations had not, however, been on a par with the increased expenditure, and it had been necessary to sell stock to make up the deficiency. The site for a Children's Home, to be erected at the seaside, had not yet been determined on. The report having been accepted, it was agreed by the committee to issue two hundred and fifty free letters granting admission to the Homes during the year, and the proceedings then came to an end.

